# Targeted Demethylation of the TGFβ1 mRNA Promotes Myoblast Proliferation via Activating the SMAD2 Signaling Pathway

**DOI:** 10.3390/cells12071005

**Published:** 2023-03-24

**Authors:** Kaiping Deng, Zhipeng Liu, Xiaodan Li, Zhen Zhang, Yixuan Fan, Qunhao Huang, Yanli Zhang, Feng Wang

**Affiliations:** 1 Institute of Sheep and Goat Science, Nanjing Agricultural University, Nanjing 210095, China; 2 Institute of Haimen Goat Industry, Nanjing Agricultural University, Nanjing 210095, China; 3 Animal Husbandry and Veterinary Station of Haimen District, Nantong 226100, China

**Keywords:** m^6^A, dCas13b, TGFβ1, cell proliferation, myoblast

## Abstract

Recent evidence suggested that N6-methyladenosine (m^6^A) methylation can determine m^6^A-modified mRNA fate and play an important role in skeletal muscle development. It was well known that transforming growth factor beta 1 (TGFβ1) is involved in a variety of cellular processes, such as proliferation, differentiation, and apoptosis. However, little is known about the m^6^A-mediated TGFβ1 regulation in myogenesis. Here, we observed an increase in endogenous TGFβ1 expression and activity during myotube differentiation. However, the knockdown of TGFβ1 inhibits the proliferation and induces cell apoptosis of myoblast. Moreover, we found that m^6^A in 5′-untranslated regions (5′UTR) of TGFβ1 promote its decay and inhibit its expression, leading to the blockage of the TGFβ1/SMAD2 signaling pathway. Furthermore, the targeted specific demethylation of TGFβ1 m^6^A using dCas13b-FTO significantly increased the TGFβ1-mediated activity of the SMAD2 signaling pathway, promoting myoblast proliferation. These findings suggest that TGFβ1 is an essential regulator of myoblast growth that is negatively regulated by m^6^A. Overall, these results highlight the critical role of m^6^A-mediated post-transcriptional regulation in myogenesis.

## 1. Introduction

Transforming growth factor beta 1 (TGFβ1) is a crucial member of TGFβ isoforms belonging to multifunctional cytokine [1]. As a polypeptide signaling molecule, TGFβ1 binds to the TGF-β receptors on the cell membrane and then transmits an extracellular signal into the cell, thereby activating the downstream SMAD pathway and controlling various cellular processes, such as cell proliferation, differentiation, and apoptosis [2]. In the skeletal muscle, TGFβ1 has been shown to have complex regulation on both muscle development and postnatal skeletal muscle mass. In low serum medium, supplementing exogenous TGFβ1 can prevent the differentiation of myoblasts by inhibiting myogenic regulatory factors expression [3,4]. However, TGFβ1 also acts as a physiological inducer of myoblast differentiation in the presence of mitogens [5]. In addition, the functional inactivation of endogenous type II TGFβ receptor, resulting in an inadequate response to TGFβ1, will impair myoblast proliferation and myogenic differentiation [6]. Similar observations also reported that TGFβ1 can activate SMAD family member 2 (SMAD2) and then promote myoblast proliferation by increasing proliferating cell nuclear antigen (PCNA) expression throughout the cell, especially in the nucleus [7,8]. Although the potential function of TGFβ1 in skeletal muscle development and regeneration has been explored in vivo and in vitro studies [9,10], its precise role in myoblasts in a specific developmental stage remains unclear. Additionally, there is little information on TGFβ1 regulation in mammal skeletal muscle development.

N6-methyladenosine (m^6^A) modification is an RNA epitranscriptomic modification mediated by methylases (such as Mettl3, Mettl14, and WTAP) and demethylases (FTO and ALKBH5), which participates in various biological processes by regulating RNA processing and metabolism [11,12,13,14]. In recent years, m^6^A has been one of the hot topics in various mammalian organ development, including embryo development [15], neurogenesis [16], adipogenesis [17], and skeletal muscle development [18]. Several studies have demonstrated that m^6^A controls myoblast proliferation and differentiation by post-transcriptional regulating the expression of key muscle-specific transcription factors [19,20]. As an important regulator of skeletal muscle development, TGFβ1 is regulated by a variety of genetic and epigenetic factors [21,22]. Recent research has shown that m^6^A can influence the epithelial-mesenchymal transformation of cancer cells by regulating the expression of TGFβ1 [23]. It has also been reported that m^6^A recognition protein Ythdf3 can recruit the PAN2-PAN3 deadenylase complex to degrade TGFβ1 mRNA during the somatic reprogramming process [24]. Furthermore, our recent report also found that silencing FTO significantly inhibits the proliferation of goat primary myoblasts (GPMs) and leads to downregulated genes being significantly enriched in the TGFβ signaling pathway [25]. These studies imply that m^6^A may affect the activity of the TGFβ signaling pathway by regulating TGFβ1 expression. However, it remains unclear whether the TGFβ1 gene in myoblast proliferation control is subject to m^6^A.

In the present study, we found that endogenous TGFβ1 expression and activity were significantly increased during the myogenic differentiation of GPMs. Then, we explored the effect of endogenous TGFβ1 expression in myoblast proliferation and the molecular mechanism underlying the regulation of m^6^A on TGFβ1 expression. Furthermore, we achieved site-specific regulation of TGFβ1 m^6^A in GPMs using dCas13b conjugated demethylase FTO. We demonstrate that targeted demethylation of TGFβ1 mRNA could upregulate its expression and promote GPMs proliferation by activating the SMAD2 signaling pathway.

## 2. Results

### 2.1. The TGFβ1 Expression Level in Myoblast Proliferation and Differentiation Phase

**Figure 1:**
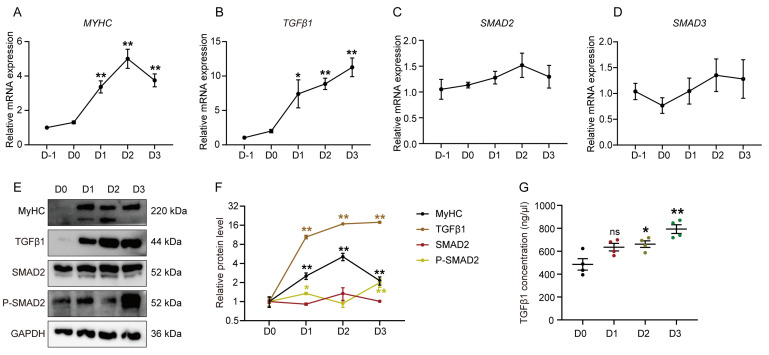
TGFβ1 expression is upregulated during myogenic differentiation. Relative mRNA expression of MyHC (**A**), TGFβ1 (**B**), SMAD2 (**C**), and SMAD3 (**D**), at days-1 (D-1), 0 (confluence [D0]), 1 (D1), 2 (D2), and 3 (D3) of differentiation. Fold changes of these genes are expressed relative to the D-1. (**E**) Western blot analysis of the protein level of MyHC, TGFβ1, SMAD2, and P-SMAD2 on D 0, 1, 2, and 3 during myogenic differentiation of GPMs. (**F**) Quantification of Western blots in (**E**). Fold change of these proteins are expressed relative to the D0. (**G**) TGFβ1 concentration in culture medium of GPMs at D 0, 1, 2, and 3 of differentiation, analyzed using ELISA. The data were obtained from at least three independent experiments. Results are expressed as mean ± SEM; * *p* < 0.05, ** *p* < 0.01, ns indicates *p* > 0.05.

To investigate the expression level of endogenous TGFβ1 and its downstream factors SMAD2/3 during myotube differentiation, GPMs were isolated and differentiated (Figure S1). As expected, MyHC expression increased significantly along with the differentiation of GPMs (Figure 1A,E). During the period (D-1–0) of myoblast proliferation, there were no significant differences in the mRNA expression level of TGFβ1, SMAD2, and SMAD3 (Figure 1B–D). During the entire period (D0–3) of myogenic differentiation, TGFβ1 expression was significantly increased (Figure 1B,E,F), while there were no significant differences in the expression levels of SMAD2 and SMAD3 (Figure 1C,D). Moreover, we assessed the endogenous TGFβ1 activity during myogenesis by detecting the phosphorylation level of SMAD2 and found that P-SMAD2 was higher at D 1 and 3 of differentiation (Figure 1E,F). Meanwhile, the TGFβ1 concentration in the culture medium also underwent an increase from D0 to D3 (Figure 1G).

### 2.2. TGFβ1 Expression Influences the Proliferation and Apoptosis of GPMs

**Figure 2:**
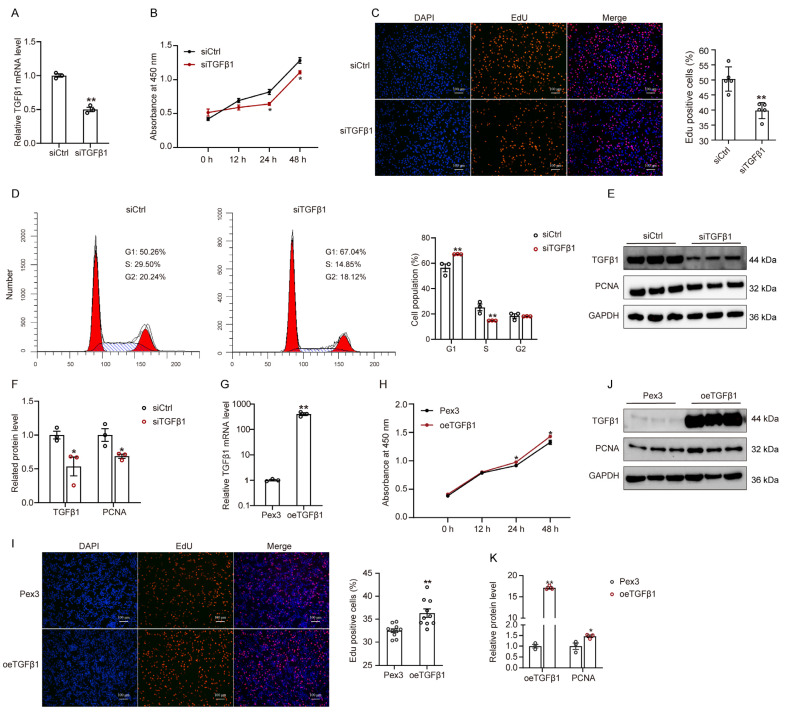
TGFβ1 promotes myoblast proliferation: (**A**–**K**) GPMs were transfected with control siRNA (siCtrl) or TGFβ1 siRNA (siTGFβ1), as well as overexpression TGFβ1 plasmid (oeTGFβ1) or empty pEX-3 vector (Pex3); (**A**,**G**) The TGFβ1 mRNA expression level was measured at 36 h post-transfection; (**B**,**H**) The viability of GPMs was detected by using CCK8 assay; (**C**,**I**) After transfection for 36 h, the cells were stained with EdU. Representative images were shown (left), scale bar, 100 μm. Quantification of the percentage of EdU-positive cells (right); (**D**) Flow cytometry analysis of cell cycle progression of cells (left), and the percentage of cells in each phase of the cell cycle was quantified; (**E**,**J**) Western blot analysis of the protein level of TGFβ1 and PCNA in cells; (**F**,**K**) Quantification of the Western blot results of (**E**,**J**). The data were obtained from at least three independent experiments. Results are expressed as mean ± SEM; * *p* < 0.05, ** *p* < 0.01.

Proof of the vital role of exogenous TGFβ1 in the proliferation, differentiation, and apoptosis of myoblast has been provided by in vitro studies [7,26]. However, the effect of endogenous TGFβ1 on myoblast growth has not been well verified. To investigate the effect of endogenous TGFβ1 on the proliferation of GPMs, we silenced (Figure 2A) or overexpressed (Figure 2G) TGFβ1 in GPMs using siRNA and pEX3-TGFβ1 plasmid, respectively. The results of CCK8 assays (Figure 2B) and EdU assays (Figure 2C) showed that TGFβ1 knockdown significantly inhibited cell proliferation. Moreover, we performed flow cytometry analysis and found that the number of cells in the G1 phase notably increased in TGFβ1 silenced cells, whereas the number of cells in the S phases significantly decreased (Figure 2D). Consistently, silencing TGFβ1 significantly decreased the protein expression of PCNA (Figure 2E,F). However, in GPMs with overexpressed TGFβ1, we observed an increase in cell proliferation (Figure 2H–K), which was contrary to the results seen in GPMs transfected TGFβ1 siRNA. These results suggest that endogenous TGFβ1 may be required for myoblast proliferation.

**Figure 3:**
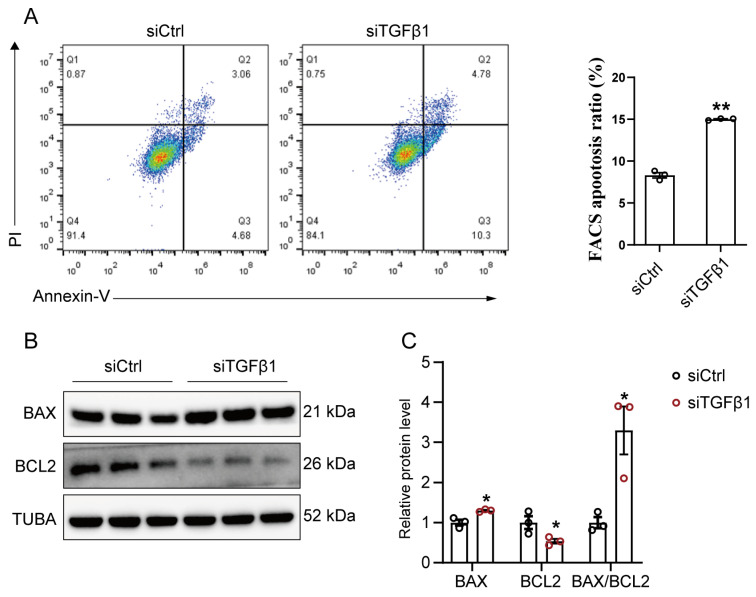
Silencing TGFβ1 promotes apoptosis of myoblast: (**A**) After transfection with control siRNA (siCtrl) or TGFβ1 siRNA (siTGFβ1) for 36 h, cell apoptosis was detected using flow cytometry; (**B**) The protein level of BAX and BCL2 in siCtrl and siTGFβ1 cells, as detected by Western blot; (**C**) Quantification of Western blots in (**B**). The data were obtained from at least three independent experiments. Results are expressed as mean ± SEM; * *p* < 0.05, ** *p* < 0.01.

To further assess the influence of silencing TGFβ1 on cellular homeostasis, we examined the levels of cell apoptosis and observed that the apoptosis rate significantly increased in siTGFβ1 cells compared to siCtrl cells (Figure 3A). Consistently, TGFβ1 knockdown significantly decreased the protein level of pro-survival protein B-cell Lymphoma-2 (BCL2), but increased the BCL-2-associated X protein (BAX) level and the ratio of BAX to BCL2 in GPMs (Figure 3B,C).

### 2.3. m^6^A Regulates the TGFβ1-Mediated SMAD2 Signaling Pathway

**Figure 4:**
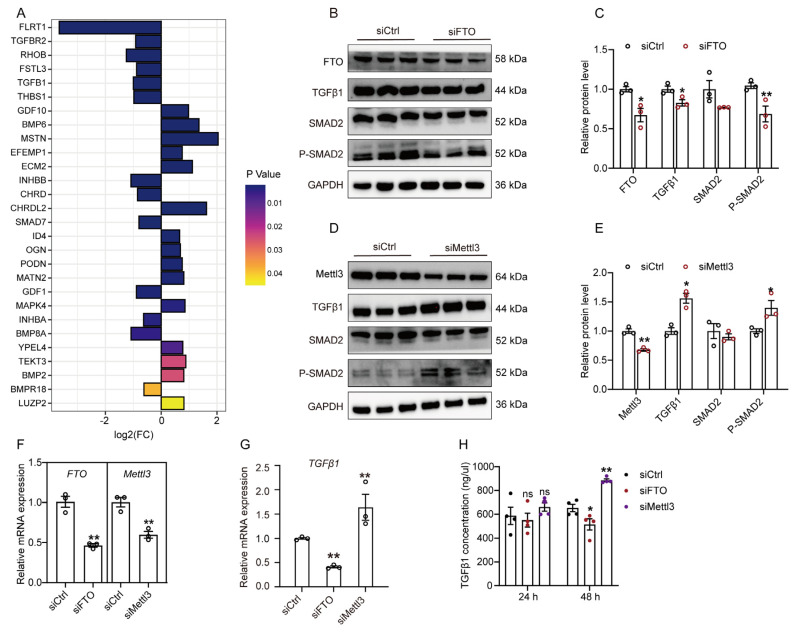
m^6^A regulates the TGFβ1-mediated SMAD2 signaling pathway: (**A**) The transcript abundance of the TGFβ pathway-related genes in differentially expressed genes between cells transfected with siCtrl and FTO siRNA (siFTO); (**B**–**E**) GPMs were treated with siFTO or Mettl3 siRNA (siMettl3); (**B**,**D**) The protein levels of TGFβ1, SMAD2, P-SMAD2 were measured at post-transfection 48 h; (**C**,**E**) Quantification of Western blots in B and D; (**F**) GPMs were treated with siFTO or Mettl3 siRNA (siMettl3), and the mRNA expression of TGFβ1 (**G**) was measured at 36 h; (**H**) After transfection for 24 h and 48 h, TGFβ1 concentration in culture medium of GPMs was measured using ELISA. The data were obtained from at least three independent experiments. Results are expressed as mean ± SEM; * *p* < 0.05, ** *p* < 0.01, ns indicates *p* > 0.05.

Based on the analysis of previous mRNA-seq datasets of FTO knockdown GPMs [25], we observed that some differentially expressed genes were significantly enriched in the TGFβ signaling pathway. Among them, TGFβR2, TGFβ1, and SMAD7 significantly downregulated (Figure 4A and Table S1), which suggests that altering m^6^A could influence the TGFβ signaling pathway. To further explore the effect of m^6^A on TGFβ1 activity, we silenced FTO and Mettl3 in GPMs. Our previous studies have shown that FTO knockdown significantly increases m^6^A levels in GPMs [27], while Mettl3 knockdown obviously decreases m^6^A levels (Unpublished). The protein level of TGFβ1 was lower in FTO-depleted cells than in control cells (Figure 4B,C). Meanwhile, by detecting the phosphorylation levels of SMAD2, we found that silencing FTO inhibited the SMAD2 activity (Figure 4B,C), which can reflect endogenous TGFβ1 activity in myoblast [28]. In contrast, Mettl3 knockdown significantly upregulated TGFβ1 protein expression and increased the phosphorylation levels of SMAD2 (Figure 4D,E). Consistently, the TGFβ1 mRNA level was significantly decreased in FTO-depleted cells, while it was increased in Mettl3-depleted cells (Figure 4F,G). In addition, we also measured TGFβ1 concentration in a culture medium by ELISA. After transfection for 48 h, FTO knockdown reduced TGFβ1 production in GPMs, while Mettl3 knockdown increased TGFβ1 production in GPMs (Figure 4H). These results suggest that m^6^A might regulate TGFβ1 expression, thereby controlling TGFβ1/SMAD2 signaling pathway in GPMs.

### 2.4. m^6^A Regulates TGFβ1 mRNA Stability in GPMs

**Figure 5:**
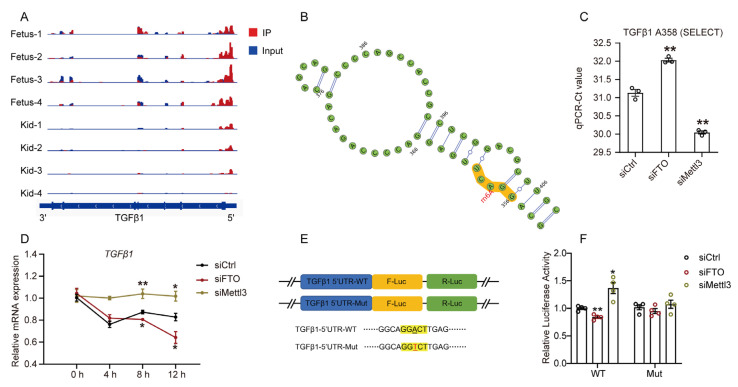
m^6^A promotes TGFβ1 mRNA decay: (**A**) The m^6^A abundance in TGFβ1 mRNA transcripts in skeletal muscle from fetuses and kids, as detected by analyzing m^6^A RIP-seq database; (**B**) m^6^A sites in 5′UTR (A358) of TGFβ1 were predicted by SRAMP (http://www.cuilab.cn/sramp, accessed on 5 February 2023); (**C**) The Ct of qRT-PCR representing SELECT results for detecting m^6^A level in the potential m^6^A site of TGFβ1 5′UTR (A358) in GPMs transfected with siFTO or siMettl3; (**D**) After treatment with actinomycin D to inhibit transcription, the mRNA stability of TGFβ1 in siCtrl, siFTO, and siMettl3 cells was quantitated by qRT-PCR; (**E**) Schematic representation of wild and mutated 5′UTR of pmirGLO vector to investigate the roles of m^6^A in 5′UTR in TGFβ1 expression; (**F**) Relative luciferase activity of wild-type or mutant pmirGLO-TGFβ1-5′UTR reporter in GPMs transfected with siFTO or siMettl3. The data were obtained from at least three independent experiments. Results are expressed as mean ± SEM; * *p* < 0.05, ** *p* < 0.01.

To further investigate the underlying mechanisms involved in the m^6^A-regulated expression of TGFβ1, we first analyzed the previous m^6^A-seq dataset of goat skeletal muscle [27] and found that TGFβ1 contains m^6^A sites in 5′UTR (Figure 5A). Moreover, we predicted and discovered an m^6^A site within 5′UTR (A358) of TGFβ1 using SRAMP online tool (Figure 5B). The result of “SELECT” showed that the m^6^A level in 5′UTR (A358) of TGFβ1 was higher in FTO-depleted cells than in control cells, but lower in Mettl3-depleted cells (Figure 5C). It is well known that altering mRNA stability is a primary way in which m^6^A mediated post-transcriptional regulation of gene expression [29]. Therefore, we investigate the influence of altering m^6^A on the TGFβ1 mRNA stability using actinomycin D. The results showed that TGFβ1 mRNA stability in FTO-depleted cells was significantly lower than that in control cells, but higher in Mettl3-depleted cells (Figure 5D). To further confirm that m^6^A within in 5′UTR of TGFβ1 regulates its expression, we generated a luciferase reporter construct of the TGFβ1 5′UTR containing the m^6^A site (Figure 5E). The wild-type or mutated pmirGLO-TGFβ1-5′UTR reporter construct was co-transfected into GPMs with siFTO or siMettl3. Then, we observed that FTO knockdown inhibited the luciferase activity of the wild-type pmirGLO-TGFβ1-5′UTR reporter, while Mettl3 knockdown increased its luciferase activity (Figure 5F). However, no significant change was observed in the luciferase activity of the mutated pmirGLO-TGFβ1-5 UTR reporter (Figure 5F). Taken together, these results suggest that m^6^A within the 5′UTR of TGFβ1 mRNA promotes its degradation, thereby inhibiting its expression in GPMs.

It is well known that Ythdf2 acted as a “reader” that recognizes m^6^A methylated mRNA and mediates m^6^A-modified mRNA stability [30]. To investigate whether Ythdf2 is involved in the regulation of TGFβ1 mRNA expression, we performed RIP-qPCR to determine if Ythdf2 protein interacts with methylated TGFβ1 mRNA in GPMs. However, we found that TGFβ1 mRNA did not bind to the Ythdf2 protein (Figure S2A). Furthermore, we observed that Ythdf2 knockdown significantly decreased the TGFβ1 mRNA level but did not affect the mRNA stability of TGFβ1 (Figure S2B,C). These results suggested that Ythdf2 is not involved in the m^6^A-regulated expression of TGFβ1 in GPMs.

### 2.5. Targeting Demethylation of TGFβ1 by dCas13b-FTO Regulates the Proliferation of GPMs

**Figure 6:**
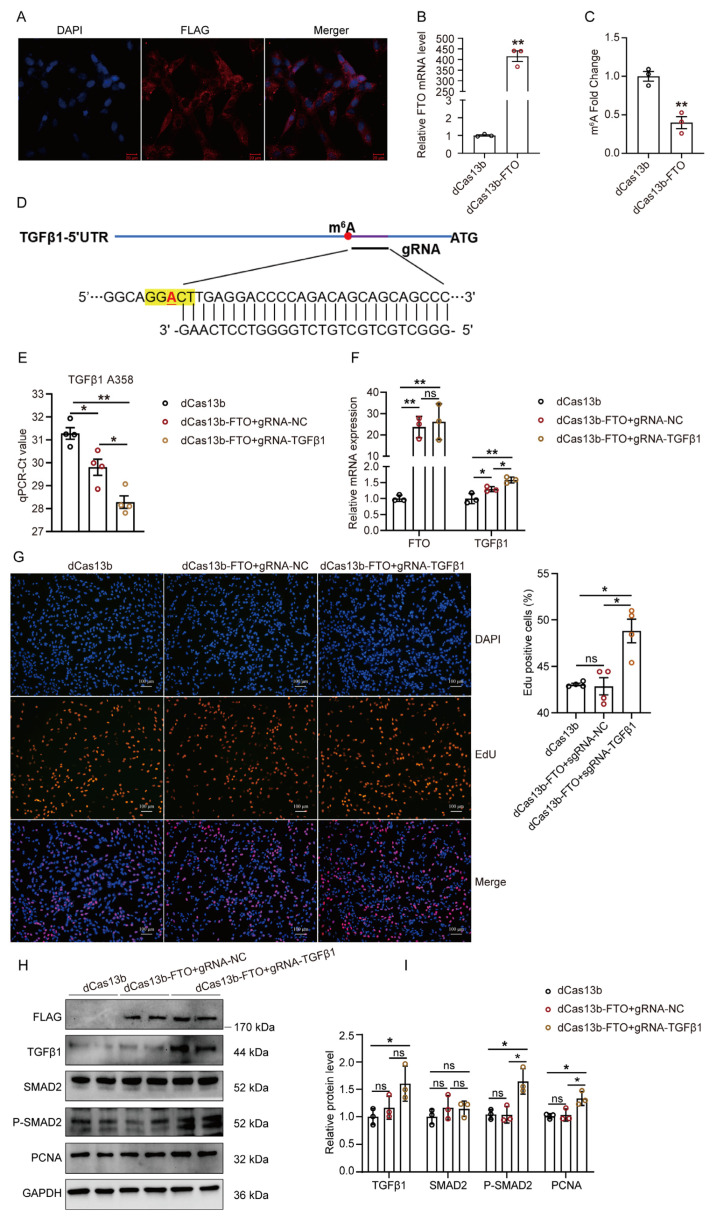
CRIPSR/dCas13b-FTO increased the expression level of TGFβ1 and promoted GPMs proliferation. After transfection with dCas13b-FTO-FLAG construct for 48 h: (**A**) Subcellular localization of the dCas13b-FTO fusion protein in GPMs was checked by confocal imaging using antibody against FLAG; and (**B**) The mRNA expression level of FTO in GPMs was detected; (**C**) The m^6^A level in GPMs transfected with dCas13b or dCas13b-FTO; (**D**) Schematic representation of positions of the m^6^A site within TGFβ1 mRNA and regions targeted by gRNA; (**E**–**I**) GPMs were transfected with dCas13b-FTO and gRNAs for 36 h; (**E**) The Ct of qRT-PCR showing SELECT results for detecting the m^6^A site in TGFβ1 at A358 in cells, with fold changes listed; (**F**) The mRNA expression of FTO and TGFβ1 in cells was measured by qRT-PCR; (**G**) The cell proliferation rate was measured by EdU assays, scale bar, 100 μm; (**H**) Western blot analysis of TGFβ1, SMAD2, P-SMAD2, and PCNA protein levels in cells; (**I**) Quantification of Western blots in H. The data were obtained from at least three independent experiments. Results are expressed as mean ± SEM; * *p* < 0.05, ** *p* < 0.01, ns indicates *p* > 0.05.

To target the demethylation of m^6^A of TGFβ1 mRNA, we first cloned the Capra hircus FTO gene (Figure S3A), and then reconstructed a fusion protein containing a catalytically dead type VI-B Cas13 enzyme and goat demethylase FTO with FLAG tag (Figure S3B). Subsequently, CRIPSR/dCas13b-FTO plasmid was transfected into GPMs. The dCas13b-FTO fusion protein was examined in GPMs using immunofluorescent staining and qRT-PCR (Figure 6A,B). As expected, the total m^6^A level of mRNA was significantly lower after transfection with dCas13b-FTO (Figure 6C). Subsequently, we designed a guide RNA that targeted the methylation region of TGFβ1 mRNA (Figure 6D and Figure S3C). The dCas13b-FTO-induced m^6^A of TGFβ1 mRNA was confirmed using “SELECT” in GPMs (Figure 6E). Additionally, we found that the expression of TGFβ1 mRNA was significantly upregulated after transfection with dCas13b-FTO and gRNA for TGFβ1 (Figure 6F).

To further investigate whether dCas13b-FTO targeting TGFβ1 controls GPMs proliferation, we measured cell proliferation using EdU assay after transfection with dCas13b-FTO combined with gRNA for TGFβ1. The results showed that the targeted demethylation of TGFβ1 mRNA increased cell proliferation in GPMs (Figure 6G). Furthermore, targeted demethylation of TGFβ1 by dCas13b-FTO increased the phosphorylation levels of P-SMAD2 and the protein levels of TGFβ1 and PCNA in GPMs (Figure 6H,I). These results confirm that targeted demethylation of TGFβ1 mRNA could promote GPMs proliferation through the TGFβ1/SMAD2 signaling pathway.

## 3. Discussion

TGFβ1 has been shown to play a particularly important role in skeletal muscle development in numerous studies [5,31,32]. However, most of these studies focused on the effects of exogenous TGFβ1 on myoblast proliferation and differentiation. Relatively few studies have explored the role of endogenous TGFβ1 in skeletal muscle development and its regulation in myogenesis. In this study, we demonstrated that endogenous TGFβ1, which is regulated by m^6^A, plays an important role in regulating myoblast proliferation. Furthermore, our data showed that dCas13b-FTO-mediated targeted demethylation of TGFβ1 mRNA positively regulates its stability and expression, thereby activating the SMAD2 signaling pathway and promoting myoblast proliferation.

Endogenous TGFβ1 expression has been found to increase in both differentiated C2C12 myoblasts and regenerating muscles [28,33]. Consistent with these findings, we observed that the content and activity of endogenous TGFβ1 were upregulated in differentiated myoblasts obtained from goat fetuses. In contrast, TGFβ1 expression has been reported to be downregulated in differentiated adult muscle progenitor cells [34]. This difference may be related to the different responses of different myoblasts to TGFβ1 [35]. Furthermore, we revealed that TGFβ1 knockdown induced the impairment of the proliferation rate in GPMs. Unlike exogenous TGFβ1, which inhibits muscle cell proliferation, endogenously synthesized TGFβ1 appears to have a positive effect on myoblast growth [6]. In addition to the inhibition of myoblast proliferation, endogenous TGFβ1 deficiency also resulted in an increase in apoptosis of GPMs. Taken together, these findings suggest that appropriate endogenous TGFβ1 may be crucial for myoblast proliferation.

The RNA modification m6A is a reversible process that regulates gene expression at the mRNA level and plays a critical role in skeletal muscle myogenesis. In recent years, more and more studies have shown that m^6^A takes part in skeletal muscle myogenesis by regulating the expression of myogenic genes, such as activin type 2 A receptors [18], MAP kinase-interacting kinase 2 [36], and myogenic marker genes (MyH2, myogenin, and MyoD1) [37,38]. Although it has been reported that Mettl3-mediated m^6^A can regulate TGFβ signaling pathway activity [39,40], the role of m^6^A in regulating TGFβ1 expression in myogenesis remains unclear. In this study, FTO knockdown was found to reduce the expression and activity of TGFβ1 in GPMs, whereas Mettl3 knockdown increased the expression and activity of TGFβ1. Furthermore, our results demonstrated that m^6^A in the 5′UTR negatively regulated TGFβ1 expression by decreasing its mRNA stability. Given that Ythdf2 is a main m^6^A reader protein involved in modulating mRNA stability [12,41], we thus consider that Ythdf2 may engage regulation in the decay of m^6^A modified TGFβ1. However, the Ythdf2 protein did not interact with the TGFβ1 transcript, which suggested that m^6^A reader protein Ythdf2 was not involved in the regulation of TGFβ1 expression via mRNA degradation. Perhaps other m^6^A reader proteins, such as Ythdf3, might be involved in the m^6^A-mediated regulation of TGBF1 expression [24]. Taken together, the results presented in this study indicate that m^6^A regulates the endogenous TGFβ1 activity in myoblasts via post-transcriptionally regulating TGFβ1.

Recently, the epi-transcriptional editing platform for m^6^A editing based on catalytically inactive Cas13 has attracted broad attention because of its applications in the revealing of functional roles of individual m^6^A in cell biology [42,43]. In this study, the fusion protein composed of dCas13b conjugated with a demethylase FTO was reconstructed. Furthermore, we specifically removed the m^6^A of TGFβ1 mRNA by the use of CRIPSR/dCas13b-FTO. The programmable system of dCas13b-FTO could lead to a decrease in m^6^A level in TGFβ1 mRNA and significantly increase its expression. Previous studies reported that TGFβ1 stimulates myoblast proliferation mainly via activating SMAD2 [7,8]. In GPMs, dCas13b-FTO can stimulate the TGFβ1/SMAD2 signaling pathway and promote cell proliferation. These findings suggest that dCas13b-FTO could target the demethylation of TGFβ1 mRNA, thereby manipulating myoblast proliferation through the TGFβ1/SMAD2 signaling pathway.

## 4. Conclusions

**Figure 7:**
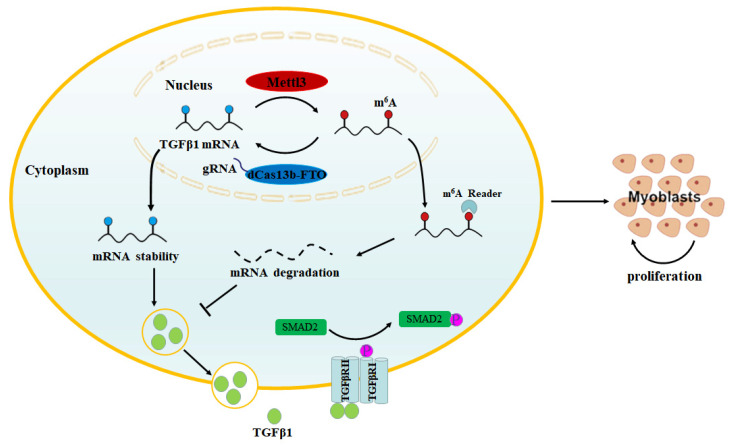
Graphical representation of the mechanisms underlying m^6^A of TGFβ1 regulating myoblast proliferation. Targeted demethylation of the TGFβ1 mRNA could increase the mRNA stability and expression of TGFβ1, and then activate the TGFβ signaling pathway, thereby promoting myoblast proliferation.

In conclusion, our findings indicate that endogenous TGFβ1 plays an important role in myoblast growth, as its deficiency inhibits myoblast proliferation and promotes cell apoptosis. In addition, we demonstrated that m^6^A regulates the TGFβ1/SMAD2 signaling pathway activity in myoblast by controlling TGFβ1 expression. Importantly, we demonstrated that targeted demethylation of TGFβ1 mRNA by dCas13b-FTO promotes myoblast proliferation by activating the SMAD2 signaling pathway (Figure 7), which highlights the potential of site-specific m^6^A editing as a tool for artificially manipulating myoblast fate.

## 5. Materials and Methods

### 5.1. Cell Culture

GPMs were isolated and purified as previously described [27]. In brief, muscle tissues were minced into pieces and digested with collagenase I (Sigma-Aldrich, Saint Louis, MO, USA) and trypsin in turn. After filtration with a 70-mm filter, the cells were collected by centrifugation at 500× *g*. Then, serial plating was performed to obtain pure GPMs. GPMs were cultured in growth media (DMEM containing 20% FBS, 10% horse serum, and 1% penicillin/streptomycin). To induce myogenic differentiation, GPMs were grown to about 80% confluence in growth media and then replaced with a differentiation medium (DMEM containing 2% horse serum and 1% penicillin/streptomycin).

### 5.2. Plasmid Construction, Small Interfering RNA (siRNA), and Cell Transfection

For TGFβ1 expression vector construction, the Capra hircus TGFβ1 coding sequence (CDS) was amplified by PCR and then subcloned into NotI and EcoRI restriction sites of the pEX3 vector (Genepharma, Shanghai, China) to generate the pEX3-TGFβ1 plasmid.

For the luciferase coding sequence, TGFβ1 5′UTR reporter vector, the DNA fragments of TGFβ1 5′UTR containing the wild-type m^6^A and mutant motifs (m^6^A was replaced by T) were synthesized, and then inserted subcloned into upstream of firefly luciferase of pmirGLO vector (Promega, Madison, WI, USA).

To generate the dCas13b-FTO fusion protein, the original PspCas13b plasmid, gRNA plasmid, and nontargeting gRNA plasmid were obtained from Addgene. The PspCas13b-FTO and gRNA-containing plasmids were constructed by TSINGKE Technologies Company (Nanjing, China). The sequences of gRNA for TGFβ1 were 5′GGGCTGCTGCTGTCTGGGGTCCTCAAG3′.

All siRNA sequences were designed and synthesized by Genepharma (Genepharma, Shanghai, China), as listed in Supplementary Table S2. For the transfection of siRNAs and plasmids, Lipofectamine 3000 transfection reagent (Life Technologies, New York, NY, USA) was used according to the manufacturer’s instructions.

### 5.3. Immunofluorescence Assay

Cells were rinsed in PBS and then fixed in 4% paraformaldehyde for 30 min at room temperature. After washing with PBS, the fixed cells were permeabilized with 0.25% Triton X-100 for 15 min. After 1 h of blocking with 3% bovine serum albumin, cells were incubated with the anti-MyHC or anti-FLAG antibodies at 4 °C overnight. The cells were subsequently washed with PBS and incubated with the secondary antibody for 1 h at room temperature. Finally, the cell nuclei were labeled with 4′,6′-diamidino-2-phenylindole (DAPI) for 10 min at room temperature, and the immunofluorescence images were analyzed using a fluorescence microscope (Zeiss LSM710 META, Jena, Germany).

### 5.4. RNA Extraction and Quantitative Real-Time PCR (qRT-PCR)

Total RNA from cell samples was extracted by using the TRIzol reagent (Invitrogen, Carlsbad, CA, USA), and cDNA was synthesized by using reverse transcription reagent kits (Vazyme, Nanjing, China). qRT-PCR was performed as described before [25]. The relative expression levels of the genes of interest were normalized to the levels of 18 S rRNA. All the primers used for qRT-PCR are listed in Supplementary Table S3.

### 5.5. mRNA Stability Analysis

After 48 h of transfection, cells were achieved by incubating actinomycin D (HY-17559; MCE, MCE, Monmouth Junction, NJ, USA) at 5 µg/mL and then were harvested at 0 h, 4 h, 8 h, and 12 h for assessing degradation. The mRNA expression level of target genes was detected by qRT-PCR, and GAPDH was used for normalization.

### 5.6. SELECT qPCR

The SELECT qPCR method was based on the previous protocol with slight modifications [44]. Briefly, 1.5 µg of total RNA was mixed with 40 nM up primer, 40 nM down primer, and 5 µM dNTP (NEB, #N0446S) in 17 µL 1× CutSmart buffer (NEB, #B7204S), and then was incubated at 90 °C, 80 °C, 70 °C, 60 °C, and 50 °C for 1 min, and then 40 °C for 6 min. Subsequently, the annealing product was further mixed with 3 µL of enzyme mixture containing 0.01 U Bst 2.0 DNA polymerase (NEB, #M0537S), 0.5 U SplintR ligase (NEB, #M0375S), and 10 nmol ATP (NEB, #P0756S), and then were incubated at 40 °C for 20 min, denatured at 80 °C for 20 min. Afterward, 2 μL of the final reaction product was used for qRT-PCR. The SELECT products of targeted sites were normalized to the threshold cycle (Ct) values of samples to their corresponding Ct values of control. All primers used in the SELECT qPCR were listed in Supplementary Table S4.

### 5.7. RNA Immunoprecipitation (RIP) Combined with Quantitative Real-Time PCR (RIP-qPCR)

RIP assay was performed by using the Imprint^®^ RNA Immunoprecipitation Kit (Sigma-Aldrich, Saint Louis, MO, USA) according to the manufacturer’s instructions. Briefly, after being transfected with Ythdf2-FLAG plasmid for 48 h, cells were lysed in lysis complete RIP lysis buffer for 10 min on ice. The supernatant was incubated with RIP buffer containing Magnetic Beads conjugated with FLAG antibody (Abcam, Cambridge, UK) or rabbit IgG at 4 °C overnight. Input and co-immunoprecipitated RNA was extracted by TRIzol reagent (Invitrogen, Carlsbad, CA, USA) and further used for qRT-PCR analyses.

### 5.8. Cell Proliferation Assay

After 24 h of transfection, cell proliferation was detected using CCK-8 (MCE, Monmouth Junction, NJ, USA) and EdU assays (Keygen Biotech, Nanjing, China) according to the manufacturer’s instructions.

### 5.9. Flow Cytometry

For cell cycle analysis, cells were harvested and then fixed overnight in 70% ethanol at −20 °C. After washing with PBS, cells were incubated in PI master mix (Keygen Biotech) for 30 min at 37 °C. Subsequently, cells were subjected to flow cytometer (Beckman Coulter, Brea, CA, USA).

For cell apoptosis analysis, cells were harvested and washed in PBS and then resuspended in ANXA5 binding buffer. After treatment with a mixture containing Annexin V-FITC and Propidium Iodide for 10 min, the cells were analyzed by flow cytometer.

### 5.10. Quantification of m^6^A Levels

The extracted total RNA from cells was purified using the GenElute mRNA Miniprep kit (MRN10, Sigma-Aldrich, Saint Louis, MO, USA) following the manufacturer’s protocols. The total m^6^A content was measured using EpiQuik m^6^A RNA Methylation Quantification Kit (P-9005-48; Epigentek, Farmingdale, NY, USA) according to the manufacturer’s instructions. About 150 ng of purified RNA was used for each sample analysis.

### 5.11. Protein Extraction and Western Blot Analysis

Cells were lysed in radioimmunoprecipitation assay (RIPA) buffer (Thermo Fisher Scientific, Waltham, MA, USA) containing a protease/phosphatase inhibitor cocktail, and the total protein was extracted and collected for Western blotting. Western blots were performed as previously described [27]. The primary antibodies used in this study are listed in Supplementary Table S5.

### 5.12. Dual-Luciferase Reporter Assays

For the dual-luciferase reporter assay, 200 ng of wild-type or mutant pmirGLO-TGFβ1-5′UTR and siFTO were co-transfected into GPMs in 24-well plates. After 48 h of transfection, the cells were harvested, and the relative luciferase activity was measured using a dual-luciferase reporter assay system (Vazyme, Nanjing, China).

### 5.13. ELISA Assay

The secretion levels of TGFβ1 in the culture medium of GPMs were measured using Goat TGFβ1 ELISA Kit (DRE-G3989c, Kmaels, Shanghai, China), following the manufacturer’s protocol. In brief, after 48 h of transfection with siFTO or siMettl3, the cell culture medium was collected. First, 50 µL of standard and samples were added to the wells, and then 100 μL of HRP-conjugate reagent was added to each well. After mixing, all wells were incubated for 60 min at 37 °C. Next, in the following sequence, washing solution, chromogen solution A, chromogen solution B, and stop solution were added and processed. Finally, the optical density (OD) value at 450 nm was measured using a microtiter plate reader.

### 5.14. Statistical Analysis

All experiments were carried out in triplicates. All results are expressed as mean ± SEM. For statistical analysis, data were analyzed by two-tailed Student’s *t* test or one-way analysis of variance (ANOVA) using SPSS software (version 24.0; SPSS, Chicago, IL, USA). For all the analyses, *p* < 0.05 was considered to be a statistically significant difference.

## Data Availability

All data generated or analyzed during this study are included in this article and its Supplementary Materials.

## Supplementary Materials

The following supporting information can be downloaded at: https://www.mdpi.com/article/10.3390/cells12071005/s1, Figure S1: Phase contrast microscopy images of GPMs at days -1 (D-1), 0 (confluence [D0]), 1 (D1), 2 (D2), and 3 (D3) of differentiation. Scale bars, 100 μm; Figure S2: Ythdf2 was not involved in m6A regulated TGFβ1 expression; Figure S3 the supplement to Figure 6; Table S1: Data of differentially expressed genes related TGFβ signaling pathway; Table S2: Details for siRNA sequence; Table S3: Details of primer sequences used for quantitative real-time PCR; Table S4: Primers used for SELECT qPCR in the present study; Table S5: Details of antibodies.
