# Supplementary material for: Targeted Demethylation of the TGFβ1 mRNA Promotes Myoblast Proliferation via Activating the SMAD2 Signaling Pathway
Source: Cells. 2023 Mar 24;12(7):1005. doi: 10.3390/cells12071005 (PMC10093215; doi:10.3390/cells12071005)
Supplement: Supplementary file 1 [file cells-12-01005-s001.zip › Supplementary Imformation-revised.pdf]

Supplementary information

Targeted Demethylation of the TGFβ1 mRNA Dromotes Myoblast Proliferation via Activating the SMAD2 Signaling Pathway

Kaiping Deng[1,2], Zhipeng Liu[1,2], Xiaodan Li [1,2], Zhen zhang [1,2], Yixuan Fan [1, 2], Qunhao Huang [3], Yanli Zhang [1,2], Feng Wang [1,2,*]

1 Institute of Sheep and Goat Science, Nanjing Agricultural University, Nanjing 210095, China

2 Institute of Haimen Goat Industry, Nanjing Agricultural University, Nanjing 210095, China

3 Animal Husbandry and Veterinary Station of Haimen District, Nantong226100, China

* Correspondence: caeet@njau.edu.cn; Tel.: +86 02584395381

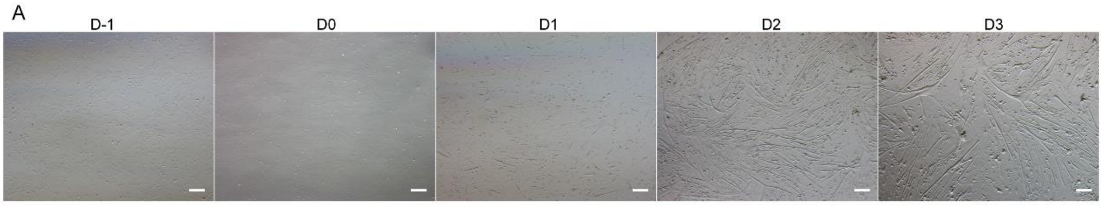

Supplementary Figure S1. Phase contrast microscopy images of GPMs at days -1 (D-1), 0 (confluence [D0]), 1 (D1), 2 (D2), and 3 (D3) of differentiation. Scale bars, 100 μm.

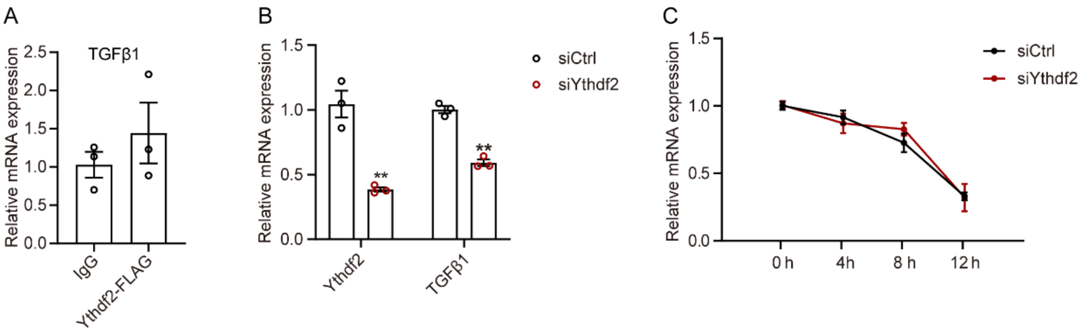

Supplementary Figure S2. Ythdf2 was not involved in m^{6}A regulated TGFβ1 expression: (**A**)Ythdf2 RIP-qPCR analysis of TGFβ1 mRNA level in GPMs transfected with YTHDF2-FLAG vector; (**B**) GPMs were treated with Ythdf2 siRNA (siYthdf2), and the mRNA expression of Ythdf2 and TGFβ1 were measured at 36 h; (**C**) After transfection with siCtrl or siYthdf2 for 36 h, cells were treated with actinomycin D for the indicated times, and the relative expression level of TGFβ1 was checked by qRT-PCR.

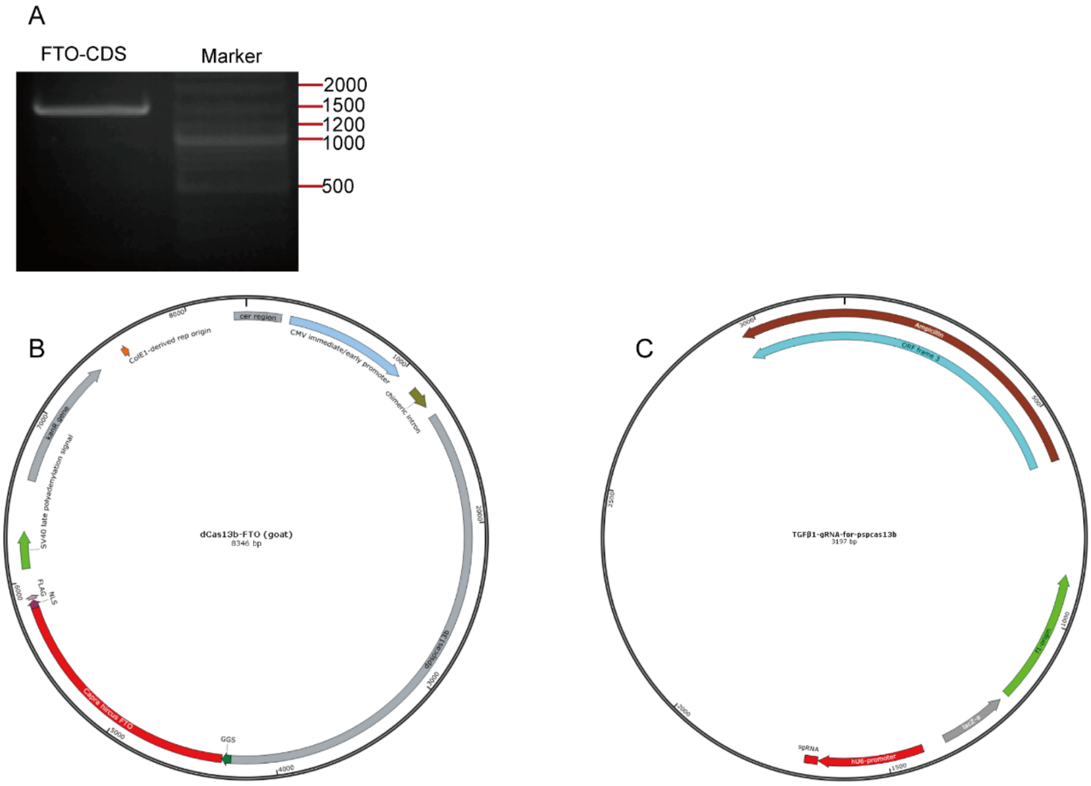

Supplementary Figure S3 the supplement to Figure 6: (**A**) The represented FTO amplification product using skeletal muscle cDNA of goat; The plasmid profiles of reconstructed CRIPSR/dCas13b-FTO plasmid (**B**) and plasmids containing gRNA targeted TGFβ1 (**C**).

Supplementary Table S2. Details for siRNA sequence.

Target	Sense (5′ to 3′)	Anti-sense (5′ to 3′)
Ctrl	UUCUCCGAACGUGUCACGUTT	ACGUGACACGUUCGGAGAATT
TGFβ1	GUGGAGCUGUACCAGAAAUTT	AUUUCUGGUACAGCUCCACTT
FTO	ACAAGCAUGGCUGUUUAUUTT	AAUAAACAGCCAUGCUUGUGC
Mettl3	GGAGAUAGAGAGCCUUCUUTT	AAGAAGGCUCUCUAUCUCCTT

Supplementary Table S3 Details of primer sequences used for quantitative real-time PCR

Items	Primer sequence (5′-3′)	Fragment size (bp)
TGFβ1	F: CTCCGTCTGCCTCCTCCTGTC<br>R: TAGGGTCCAAGGTGCTCAGTCAAG	147
Ythdf2	F: CAGGCATCAGTAGGGCAACA<br>R: GGACCGCAACTTCTCCAAGA	228
SMAD2	F: TGGACCACCGCTTTTGGTAA<br>R: CCATCTAGAGACCTTGGTATGGT	272
SMAD3	F: GCCCACAGGTTCTCAAGGAA<br>R: TTCAACATCGGGCTGTGAGT	285
FTO	F: AACGAGAGCGCGAAGCTAAG<br>R: CCGATGAGGATGCGAGAGAC	274
Mettl3	F: GGTCTGGCCTCTTCCACATC<br>R: CTTGAACTTGAGCCCGACCT<br>R: GAAAGTCGTTGTGGGCGGTT	229
MyHC	F: CTGTCCAAGTTCCGCAAGGT<br>R: GAGCTTCGTTGCACCCTCAA<br>R: TTCATGGTGGCTGCATCAGT	182
18S rRNA	F: GTGATGGGGATCGGGGATTG	116

	R: GTAGCGACGGGCGGTGTGTA		
GAPDH	R: CCGTTCGACAGATAGCCGTAA<br>F: AGGATCTCGCTCCTGGAAGAT	296	
qPCR-SELECT	R: ATGCAGCGACTCAGCCTCTG<br>F: TAGCCAGTACCGTAGTGCGTG		

Supplementary Table S4. Primers used for SELECT qPCR in the present study

SELECT	UP (5′ to 3′)	DOWN (5′ to 3′)
TGFβ1-A358	tagccagtaccgtagtgcgtgtgctgtctggggtcc tcaag	**5phos**/cctgcctcgcggagcaacgccagaggctgagt cgctgcat

Supplementary Table S5. Details of antibodies

Antibodies	Cat No.	Source	Dilution of WB
MyHC	Ab11083	Abcam	1:3000
TGFβ1	Ab215715	Abcam	1:1000
PCNA	ab18197	Abcam	1:1000
BAX	50599-2-Ig	Proteintech	1:5000
BCL2	12789-1-AP	Proteintech	1:1000
FTO	bs-7056R	Bioss	1:1000
METTL3	15073-1-AP	Proteintech	1:1000
Smad2	AF6497	Affinity	1:1000
Phospho-Smad2 (Ser467)	AF3449	Affinity	1:1000
FLAG	T0003	Affinity	1:5000
TUBA	11224-1-AP	Proteintech	1:5000
GADPH	60004-1-IG	Proteintech	1:8000
Goat anti-Rabbit IgG (H+L) Secondary Antibody, HRP	31460	Invitrogen	1:10000
HRP-conjugated Affinipure Goat Anti-Mouse IgG(H+L)	SA00001-1	Proteintech	1:8000
