# Supplementary figures and images for: Targeted Demethylation of the TGFβ1 mRNA Promotes Myoblast Proliferation via Activating the SMAD2 Signaling Pathway
Source: Cells. 2023 Mar 24;12(7):1005. doi: 10.3390/cells12071005 (PMC10093215; doi:10.3390/cells12071005)

Fig. 1E

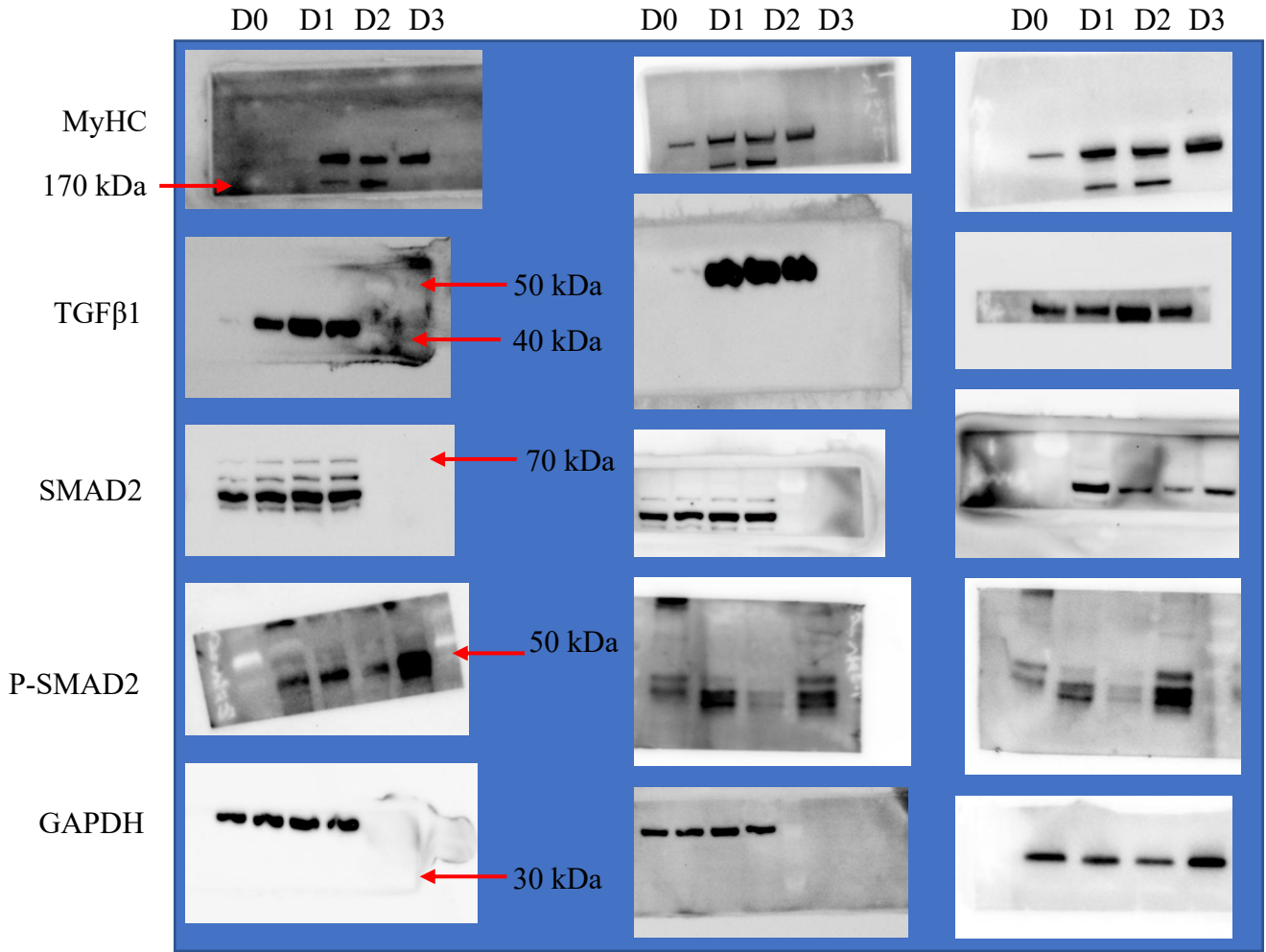

Fig. 2E

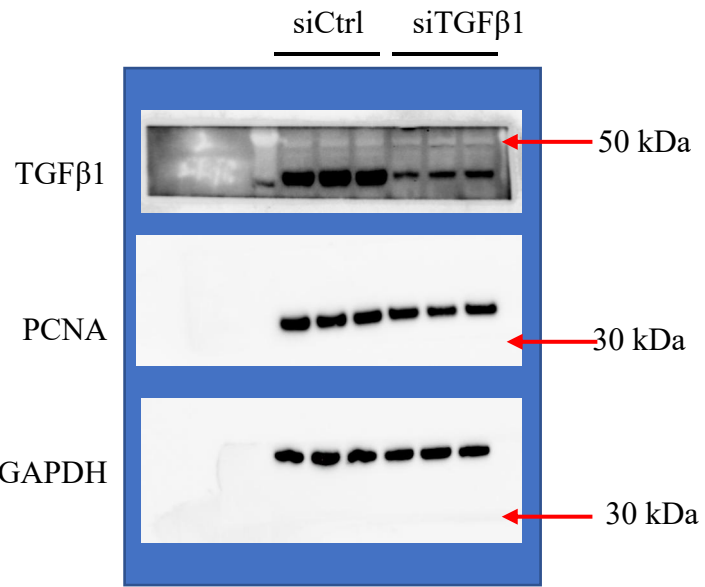

Fig. 2J

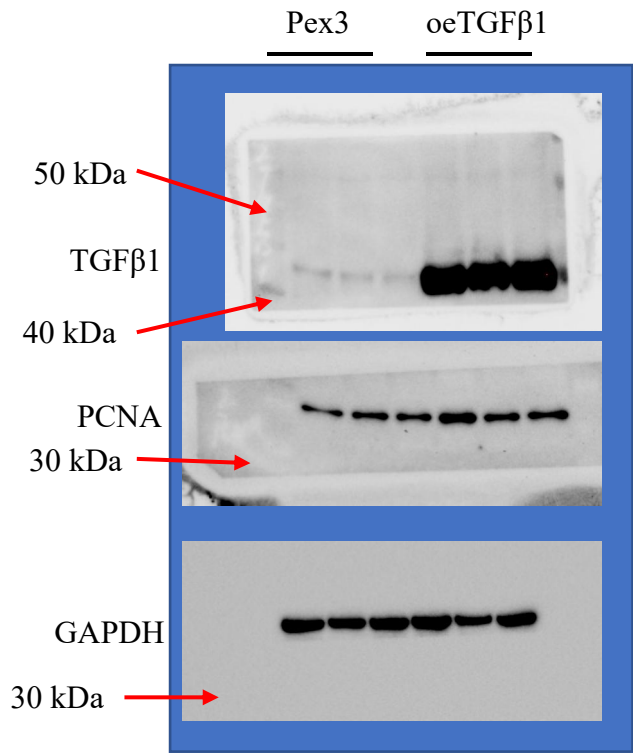

Fig. 3B

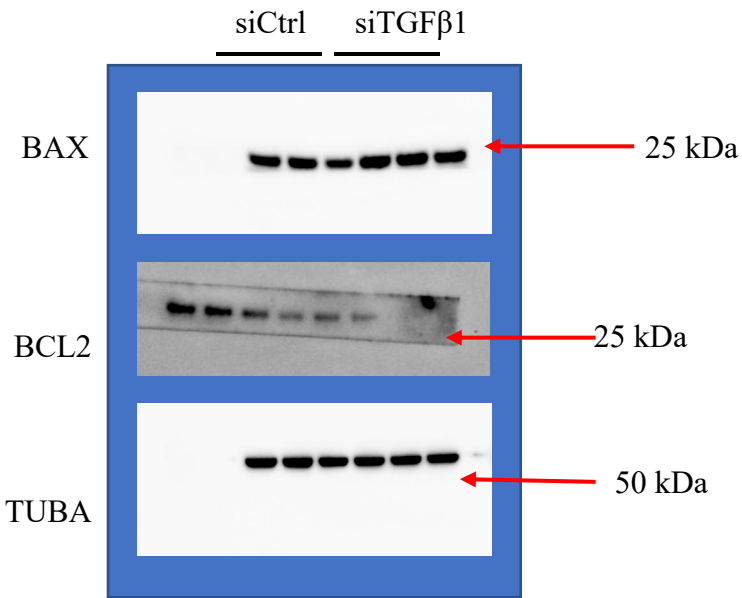

Fig. 4C

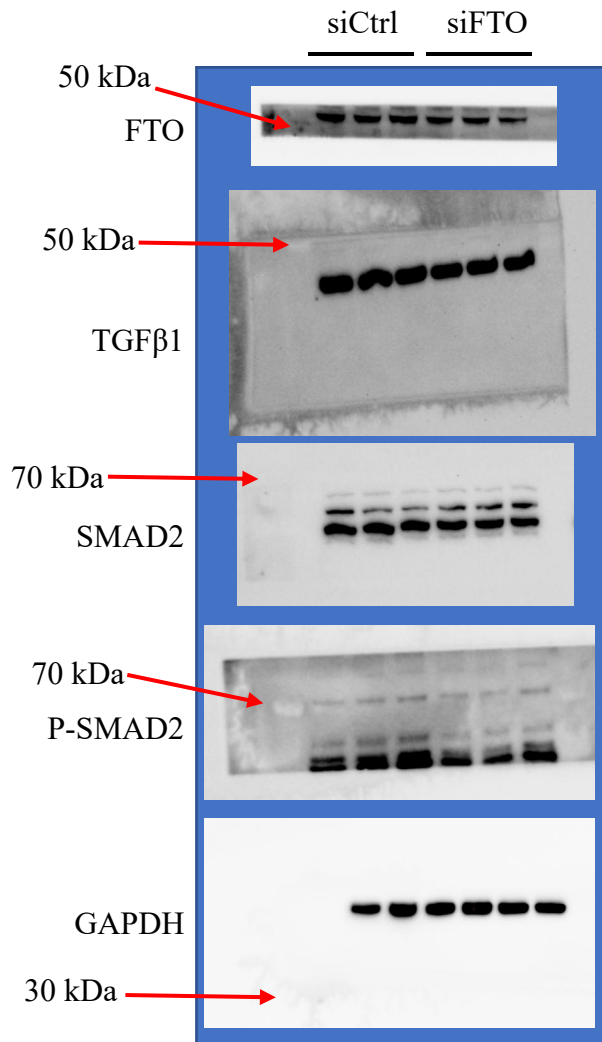

Fig. 4E

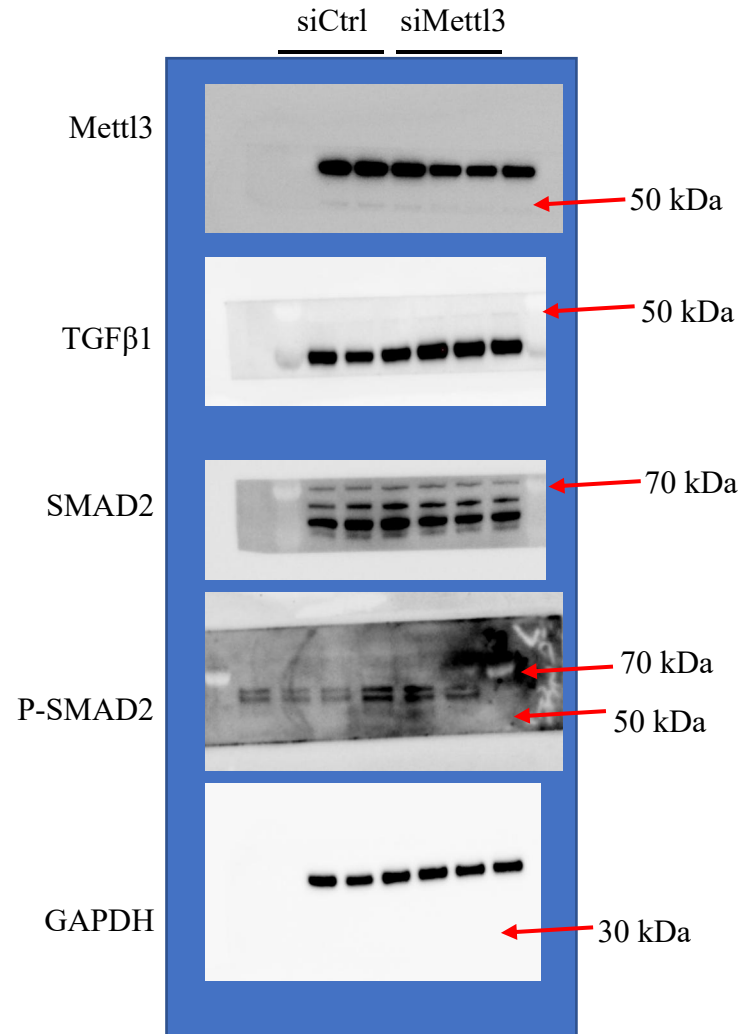

Fig 6H

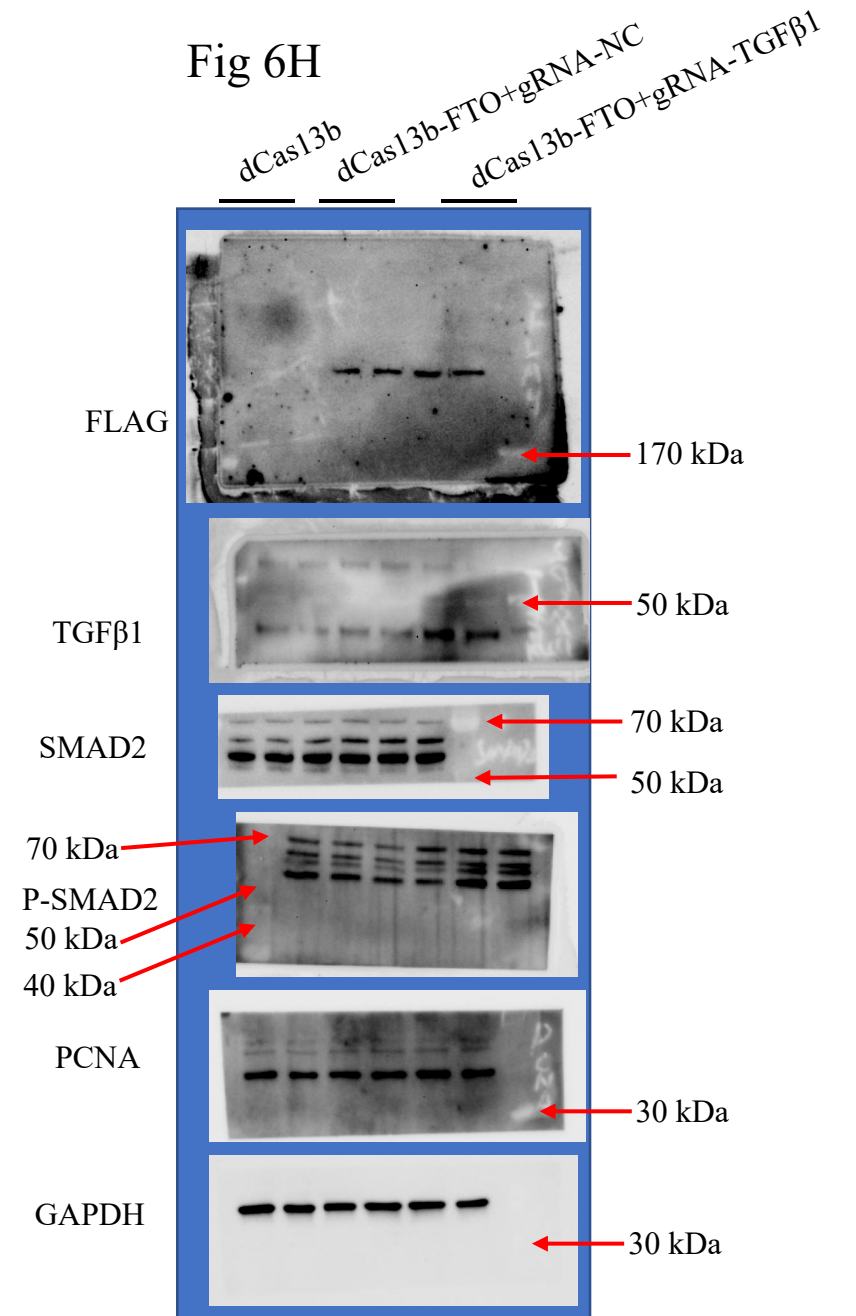

Supplement: Supplementary file 1 [file cells-12-01005-s001.zip › Western blot original picture-revised.pdf]
